# Effects of the COVID-19 pandemic on medical students: a multicenter quantitative study

**DOI:** 10.1186/s12909-020-02462-1

**Published:** 2021-01-06

**Authors:** Aaron J. Harries, Carmen Lee, Lee Jones, Robert M. Rodriguez, John A. Davis, Megan Boysen-Osborn, Kathleen J. Kashima, N. Kevin Krane, Guenevere Rae, Nicholas Kman, Jodi M. Langsfeld, Marianne Juarez

**Affiliations:** 1 Department of Emergency Medicine, University of California San Francisco School of Medicine, San Francisco General Hospital, 1001 Potrero Avenue, Building 5, Room #6A4, San Francisco, California 94110 USA; 2 University of California San Francisco School of Medicine, San Francisco, California USA; 3 Clinical Emergency Medicine, University of California Irvine School of Medicine, Irvine, CA USA; 4 University of Illinois College of Medicine, Chicago, IL USA; 5 Deming Department of Medicine, Tulane University School of Medicine, New Orleans, Louisiana USA; 6 Basic Science Education, Tulane University School of Medicine, New Orleans, Louisiana USA; 7 Emergency Medicine, Ohio State College of Medicine, Columbus, OH USA; 8 Department of Science Education, Donald and Barbara Zucker School of Medicine at Hofstra/Northwell, Hempstead, New York USA

**Keywords:** Undergraduate medical education, COVID-19 pandemic, Medical student anxiety

## Abstract

**Background:**

The COVID-19 pandemic disrupted the United States (US) medical education system with the necessary, yet unprecedented Association of American Medical Colleges (AAMC) national recommendation to pause all student clinical rotations with in-person patient care. This study is a quantitative analysis investigating the educational and psychological effects of the pandemic on US medical students and their reactions to the AAMC recommendation in order to inform medical education policy.

**Methods:**

The authors sent a cross-sectional survey via email to medical students in their clinical training years at six medical schools during the initial peak phase of the COVID-19 pandemic. Survey questions aimed to evaluate students’ perceptions of COVID-19’s impact on medical education; ethical obligations during a pandemic; infection risk; anxiety and burnout; willingness and needed preparations to return to clinical rotations.

**Results:**

Seven hundred forty-one (29.5%) students responded. Nearly all students (93.7%) were not involved in clinical rotations with in-person patient contact at the time the study was conducted. Reactions to being removed were mixed, with 75.8% feeling this was appropriate, 34.7% guilty, 33.5% disappointed, and 27.0% relieved.

Most students (74.7%) agreed the pandemic had significantly disrupted their medical education, and believed they should continue with normal clinical rotations during this pandemic (61.3%). When asked if they would accept the risk of infection with COVID-19 if they returned to the clinical setting, 83.4% agreed.

Students reported the pandemic had moderate effects on their stress and anxiety levels with 84.1% of respondents feeling at least somewhat anxious. Adequate personal protective equipment (PPE) (53.5%) was the most important factor to feel safe returning to clinical rotations, followed by adequate testing for infection (19.3%) and antibody testing (16.2%).

**Conclusions:**

The COVID-19 pandemic disrupted the education of US medical students in their clinical training years. The majority of students wanted to return to clinical rotations and were willing to accept the risk of COVID-19 infection. Students were most concerned with having enough PPE if allowed to return to clinical activities.

**Supplementary Information:**

The online version contains supplementary material available at 10.1186/s12909-020-02462-1.

## Background

The COVID-19 pandemic has tested the limits of healthcare systems and challenged conventional practices in medical education. The rapid evolution of the pandemic dictated that critical decisions regarding the training of medical students in the United States (US) be made expeditiously, without significant input or guidance from the students themselves. On March 17, 2020, for the first time in modern US history, the Association of American Medical Colleges (AAMC), the largest national governing body of US medical schools, released guidance recommending that medical students immediately pause all clinical rotations to allow time to obtain additional information about the risks of COVID-19 and prepare for safe participation in the future. This decisive action would also conserve scarce resources such as personal protective equipment (PPE) and testing kits; minimize exposure of healthcare workers (HCWs) and the general population; and protect students’ education and wellbeing [1].

A similar precedent was set outside of the US during the SARS-CoV1 epidemic in 2003, where an initial cluster of infection in medical students in Hong Kong resulted in students being removed from hospital systems where SARS surfaced, including Hong Kong, Singapore and Toronto [2, 3]. Later, studies demonstrated that the exclusion of Canadian students from those clinical environments resulted in frustration at lost learning opportunities and students’ inability to help [3]. International evidence also suggests that medical students perceive an ethical obligation to participate in pandemic response, and are willing to participate in scenarios similar to the current COVID-19 crisis, even when they believe the risk of infection to themselves to be high [4–6].

The sudden removal of some US medical students from educational settings has occurred previously in the wake of local disasters, with significant academic and personal impacts. In 2005, it was estimated that one-third of medical students experienced some degree of depression or post-traumatic stress disorder (PTSD) after Hurricane Katrina resulted in the closure of Tulane University School of Medicine [7].

Prior to the current COVID-19 pandemic, we found no studies investigating the effects of pandemics on the US medical education system or its students. The limited pool of evidence on medical student perceptions comes from two earlier global coronavirus surges, SARS and MERS, and studies of student anxiety related to pandemics are also limited to non-US populations [3, 8, 9]. Given the unprecedented nature of the current COVID-19 pandemic, there is concern that students may be missing out on meaningful educational experiences and months of clinical training with unknown effects on their current well-being or professional trajectory [10].

Our study, conducted during the initial peak phase of the COVID-19 pandemic, reports students’ perceptions of COVID-19’s impact on: medical student education; ethical obligations during a pandemic; perceptions of infection risk; anxiety and burnout; willingness to return to clinical rotations; and needed preparations to return safely. This data may help inform policies regarding the roles of medical students in clinical training during the current pandemic and prepare for the possibility of future pandemics.

## Methods

We conducted a cross-sectional survey during the initial peak phase of the COVID-19 pandemic in the United States, from 4/20/20 to 5/25/20, via email sent to all clinically rotating medical students at six US medical schools: University of California San Francisco School of Medicine (San Francisco, CA), University of California Irvine School of Medicine (Irvine, CA), Tulane University School of Medicine (New Orleans, LA), University of Illinois College of Medicine (Chicago, Peoria, Rockford, and Urbana, IL), Ohio State University College of Medicine (Columbus, OH), and Zucker School of Medicine at Hofstra/Northwell (Hempstead, NY). Traditional undergraduate medical education in the US comprises 4 years of medical school with 2 years of primarily pre-clinical classroom learning followed by 2 years of clinical training involving direct patient care. Study participants were defined as medical students involved in their clinical training years at whom the AAMC guidance statement was directed. Depending on the curricular schedule of each medical school, this included intended graduation class years of 2020 (graduating 4th year student), 2021 (rising 4th year student), and 2022 (rising 3rd year student), exclusive of planned time off. Participating schools were specifically chosen to represent a broad spectrum of students from different regions of the country (West, South, Midwest, East) with variable COVID-19 prevalence. We excluded medical students not yet involved in clinical rotations. This study was deemed exempt by the respective Institutional Review Boards.

We developed a survey instrument modeled after a survey used in a previously published peer reviewed study evaluating the effects of the COVID-19 pandemic on Emergency Physicians, which incorporated items from validated stress scales [11]. The survey was modified for use in medical students to assess perceptions of the following domains: perceived impact on medical student education; ethical beliefs surrounding obligations to participate clinically during the pandemic; perceptions of personal infection risk; anxiety and burnout related to the pandemic; willingness to return to clinical rotations; and preparation needed for students to feel safe in the clinical environment. Once created, the survey underwent an iterative process of input and review from our team of authors with experience in survey methodology and psychometric measures to allow for optimization of content and validity. We tested a pilot of our preliminary instrument on five medical students to ensure question clarity, and confirm completion of the survey in approximately 10 min. The final survey consisted of 29 Likert, yes/no, multiple choice, and free response questions. Both medical school deans and student class representatives distributed the survey via email, with three follow-up emails to increase response rates. Data was collected anonymously.

For example, to assess the impact on students’ anxiety, participants were asked, “How much has the COVID-19 pandemic affected your stress or anxiety levels?” using a unipolar 7-point scale (1 = not at all, 4 = somewhat, 7 = extremely). To assess willingness to return to clinical rotations, participants were asked to rate on a bipolar scale (1 = strongly disagree, 2 = disagree, 3 = somewhat disagree, 4 = neither disagree nor agree, 5 = somewhat agree, 6 = agree, and 7 = strongly agree) their agreement with the statement: “to the extent possible, medical students should continue with normal clinical rotations during this pandemic.” (Survey Instrument, Supplemental Table 1).

Survey data was managed using Qualtrics hosted by the University of California, San Francisco. For data analysis we used STATA v15.1 (Stata Corp, College Station, TX). We summarized respondent characteristics and key responses as raw counts, frequency percent, medians and interquartile ranges (IQR). For responses to bipolar questions, we combined positive responses (somewhat agree, agree, or strongly agree) into an agreement percentage. To compare differences in medians we used a signed rank test with *p* value < 0.05 to show statistical difference. In a secondary analysis we stratified data to compare questions within key domains amongst the following sub-groups: female versus male, graduation year, local community COVID-19 prevalence (high, medium, low), and students on clinical rotations with in-person patient care. This secondary analysis used a chi square test with *p* value < 0.05 to show statistical difference between sub-group agreement percentages.

## Results

Of 2511 students contacted, we received 741 responses (29.5% response rate). Of these, 63.9% of respondents were female and 35.1% were male, with 1.0% reporting a different gender identity; 27.7% of responses came from the class of 2020, 53.5% from the class of 2021, and 18.7% from the class of 2022. (Demographics, Table 1).

**Table 1:** Demographics

	**20–24**	**25–29**	**30–34**	**35–39**	**40–44**		**45+**
Age *n* (%)	87 (12.5%)	493 (70.9%)	98 (14.1%)	14 (2.0%)	1 (0.1%)		2 (0.3%)
	**Male**	**Female**	**Trans Male**	**Trans Female**	**Genderqueer/ Nonbinary**		**Other**
Gender^a^ *n* (%)	242 (35.1%)	443 (63.9%)	1 (0.1%)	0 (0.0%)	8 (1.2%)		2 (0.3%)
	**African-American**	**Asian**	**Hispanic/Latinx**	**Native American/ American Indian**	**Native Hawaiian/ Pacific Islander**	**White**	**Other**
Race/Ethnicity^a^ *n* (%)	42 (6.1%)	199 (29.1%)	77 (11.2%)	5 (0.7%)	1 (0.1%)	408 (59.6%)	36 (5.3%)
		**2020**		**2021**		**2022**	
Graduation Year^b^ *n* (%)		193 (27.8%)		372 (53.5%)		130 (18.7%)	
	**West**	**Midwest**		**South**		**East**	
US Region *n* (%)	290 (41.8%)	114 (16.4%)		176 (25.3%)		110 (15.9%)	

^a^allowed students to select more than one response ^b^excluding any planned time off

Most student respondents (74.9%) had a clinical rotation that was cut short or canceled due to COVID-19 and 93.7% reported not being involved in clinical rotations with in-person patient contact at the time of the study. Regarding students’ perceptions of cancelled rotations (allowing for multiple reactions), 75.8% felt this was appropriate, 34.7% felt guilty for not being able to help patients and colleagues, 33.5% felt disappointed, and 27.0% felt relieved.

**Fig. 1:**
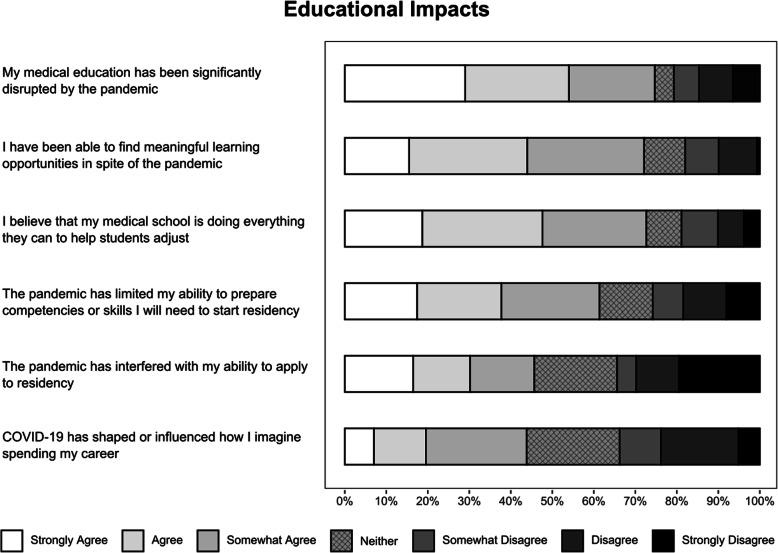
Perceived educational impacts of the COVID-19 pandemic on medical students

Most students (74.7%) agreed that their medical education had been significantly disrupted by the pandemic. Students also felt they were able to find meaningful learning experiences during the pandemic (72.1%). Free response examples included: taking a novel COVID-19 pandemic elective course, telehealth patient care, clinical rotations transitioned to virtual online courses, research or education electives, clinical and non-clinical COVID-19-related volunteering, and self-guided independent study electives. Students felt their medical schools were doing everything they could to help students adjust (72.7%). Overall, respondents felt the pandemic had interfered with their ability to develop skills needed to prepare for residency (61.4%), though fewer (45.7%) felt it had interfered with their ability to apply to residency. (Educational Impact, Fig. 1).

**Fig. 2:**
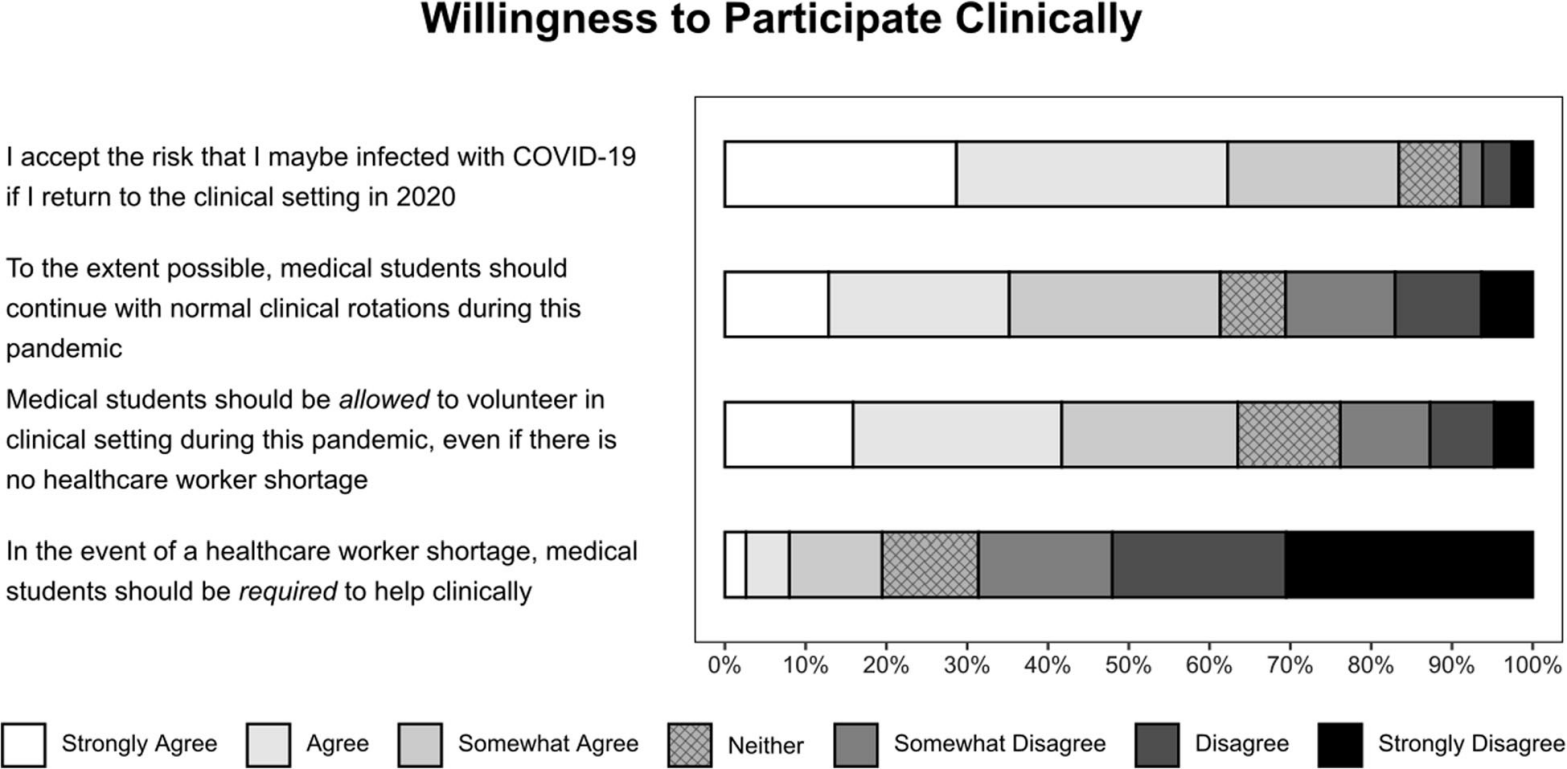
Willingness to participate clinically during the COVID-19 pandemic

A majority of medical students agreed they should be allowed to continue with normal clinical rotations during this pandemic (61.3%). Most students agreed (83.4%) that they accepted the risk of being infected with COVID-19, if they returned. When asked if students should be *allowed* to volunteer in clinical settings even if there is not a healthcare worker (HCW) shortage, 63.5% agreed; however, in the case of a HCW shortage only 19.5% believed students should be *required* to volunteer clinically. (Willingness to Participate Clinically, Fig. 2).

**Fig. 3:**
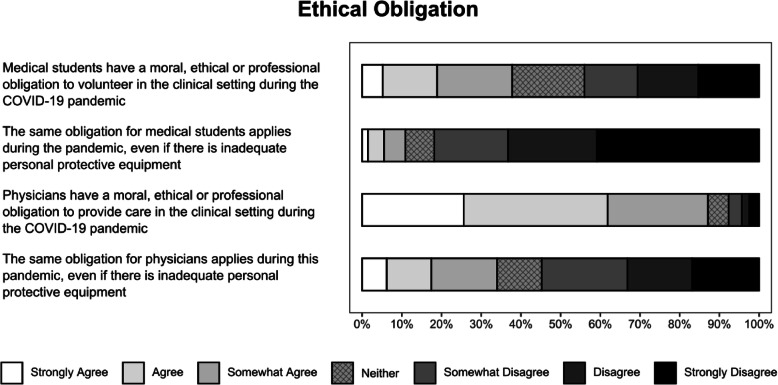
Ethical obligation to volunteer during the COVID-19 pandemic

When asked if they perceived a moral, ethical, or professional obligation for medical students to help, 37.8% agreed that medical students have such an obligation during the current pandemic. This is in contrast to their perceptions of physicians: 87.1% of students agreed with a physician obligation to help during the COVID-19 pandemic. For both groups, students were asked if this obligation persisted without adequate PPE: only 10.9% of students believed medical students had this obligation, while 34.0% agreed physicians had this obligation. (Ethical Obligation, Fig. 3).

Given the assumption that there will not be a COVID-19 vaccine until 2021, students felt the single most important factor in a safe return to clinical rotations was having access to adequate PPE (53.3%), followed by adequate testing for infection (19.3%) and antibody testing for possible immunity (16.2%). Few students (5%) stated that nothing would make them feel comfortable until a vaccine is available. On a 1–7 scale (1 = not at all, 4 = somewhat, 7 = extremely), students felt somewhat prepared to use PPE during this pandemic in the clinical setting, median = 4 (IQR 4,6), and somewhat confident identifying symptoms most concerning for COVID-19, median = 4 (IQR 4,5). Students preferred to learn about PPE via video demonstration (76.7%), online modules (47.7%), and in-person or Zoom style conferences (44.7%).

Students believed they were likely to contract COVID-19 in general (75.6%), independent of a return to the clinical environment. Most respondents believed that missing some school or work would be a likely outcome (90.5%), and only a minority of students believed that hospitalization (22.1%) or death (4.3%) was slightly, moderately, or extremely likely.

On a 1–7 scale (1 = not at all, 4 = somewhat, and 7 = extremely), the median (IQR) reported effect of the COVID-19 pandemic on students’ stress or anxiety level was 5 (4, 6) with 84.1% of respondents feeling at least somewhat anxious due to the pandemic. Students’ perceived emotional exhaustion and burnout *before the pandemic* was a median = 2 (IQR 2,4) and *since the pandemic started* a median = 4 (IQR 2,5) with a median difference Δ = 2, *p* value < 0.001.

Secondary analysis of key questions revealed statistical differences between sub-groups. Women were significantly more likely than men to agree that the pandemic had affected their anxiety. Several significant differences existed for the class of 2020 when compared to the classes of 2021 and 2022: they were less likely to report disruptions to their education, to prefer to return to rotations, and to report an effect on anxiety. There were no significant differences with students who were still involved with in-person patient care compared with those who were not. In comparing areas with high COVID-19 prevalence at the time of the survey (New York and Louisiana) with medium (Illinois and Ohio) and low prevalence (California), students were less likely to report that the pandemic had disrupted their education. Students in low prevalence areas were most likely to agree that medical students should return to rotations. There were no differences between prevalence groups in accepting the risk of infection to return, or subjective anxiety effects. (Stratification, Table 2).

**Table 2:** Stratification

Agreement with the following statements	Total n	My medical education has been significantly disrupted by the pandemic		To the extent possible, medical students should continue with normal clinical rotations during this pandemic		I accept the risk that I may be infected with COVID-19 if I return to the clinical setting in 2020		How much has the COVID-19 pandemic affected your stress or anxiety levels?	
		# agreeing/# of Respondents (%)	*p*	# agreeing/# of Respondents (%)	*p*	# agreeing/# of Respondents (%)	*p*	# agreeing/# of Respondents (%)	*p*
All respondents	741	520/696 (74.7)		425/693 (61.3)		579/694 (83.4)		619/736 (84.1)	
Gender									
Female	443	332/442 (75.1)	0.78	273/440 (62.1)	0.87	375/440 (85.2)	0.25	394/439 (89.8)	**< 0.001**
Male	243	184/242 (76.0)		148/241 (61.4)		198/242 (81.8)		177/242 (73.1)	
Graduation Class									
2020	193	74/191 (38.7)	**< 0.001**	92/190 (48.4)	**< 0.001**	167/190 (87.9)	0.049	146/191 (76.4)	**0.003**
2021	372	332/371 (89.5)		240/371 (64.7)		299/371 (80.6)		320/369 (86.7)	
2022	130	111/130 (85.4)		92/128 (71.9)		112/129 (86.8)		114/130 (87.7)	
COVID-19 prevalence									
High (NY,LA)	288	196/286 (68.5)	**< 0.001**	169/285 (59.3)	**0.019**	238/285 (83.5)	0.82	239/288 (83.0)	0.58
Medium (OH,IL)	111	98/111 (88.3)		58/110 (52.7)		91/111 (82.0)		96/110 (87.3)	
Low (CA)	292	220/292 (75.3)		195/291 (67.0)		246/291 (84.5)		242 /288 (84.0)	
Were on clinical rotations at time of study									
Yes	47	34/41 (82.9)	0.21	31/41 (75.6)	0.053	35/41 (85.4)	0.73	39/47 (83.0)	0.83
No	694	486/655 (74.2)		394/652 (60.4)		544/653 (83.3)		580/689 (84.2)	

## Discussion

The COVID-19 pandemic has fundamentally transformed education at all levels - from preschool to postgraduate. Although changes to K-12 and college education have been well documented [12, 13], there have been very few studies to date investigating the effects of COVID-19 on undergraduate medical education [14]. To maintain the delicate balance between student safety and wellbeing, and the time-sensitive need to train future physicians, student input must guide decisions regarding their roles in the clinical arena. Student concerns related to the pandemic, paired with their desire to return to rotations despite the risks, suggest that medical students may take on emotional burdens as members of the patient care team even when not present in the clinical environment. This study offers insight into how best to support medical students as they return to clinical rotations, how to prepare them for successful careers ahead, and how to plan for their potential roles in future pandemics.

Previous international studies of medical student attitudes towards hypothetical influenza-like pandemics demonstrated a willingness (80%) [4] and a perceived ethical obligation to volunteer (77 and 70%), despite 40% of Canadian students in one study perceiving a high likelihood of becoming infected [5, 6]. Amidst the current COVID-19 pandemic, our participants reported less agreement with a medical student ethical *obligation* to volunteer in the clinical setting at 37.8%, but believed in a higher likelihood of becoming infected at 75.6%. Their willingness to be *allowed* to volunteer freely (63.5%) may suggest that the stresses of an ongoing pandemic alter students’ perceptions of the ethical requirement more than their willingness to help. Students overwhelmingly agreed that physicians had an ethical obligation to provide care during the COVID-19 pandemic (87.1%), possibly reflecting how they view the ethical transition from student to physician, or differences between paid professionals and paying for an education.

At the time our study was conducted, there were widespread concerns for possible HCW shortages. It was unclear whether medical students would be called to volunteer when residents became ill, or even graduate early to start residency training immediately (as occurred at half of schools surveyed). This timing allowed us to capture a truly unique perspective amongst medical students, a majority of whom reported increased anxiety and burnout due to the pandemic. At the same time, students felt that their medical schools were doing everything possible to support them, perhaps driven by virtual town halls and daily communication updates.

Trends in secondary analysis show important differences in the impacts of the pandemic. Women were more likely to report increased anxiety as compared to men, which may reflect broader gender differences in medical student anxiety [15] but requires more study to rule out different pandemic stresses by gender. Graduating medical students (class of 2020) overall described less impact on medical education and anxiety, a decreased desire to return to rotations, but equal acceptance of the risk of infection in clinical settings, possibly reflecting a focus on their upcoming intern year rather than the remaining months of undergraduate medical education. Since this class’s responses decreased overall agreement on these questions, educational impacts and anxiety effects may have been even greater had they been assessed further from graduation. Interestingly, students from areas with high local COVID-19 prevalence (New York and Louisiana) reported a less significant effect of the pandemic on their education, a paradoxical result that may indicate that medical student tolerance for the disruptions was greater in high-prevalence areas, as these students were removed at the same, if not higher, rates as their peers. Our results suggest that in future waves of the current pandemic or other disasters, students may be more patient with educational impacts when they have more immediate awareness of strains on the healthcare system.

A limitation of our study was the survey response rate, which was anticipated given the challenges students were facing. Some may not have been living near campus; others may have stopped reading emails due to early graduation or limited access to email; and some would likely be dealing with additional personal challenges related to the pandemic. We attempted to increase response rates by having the study sent directly from medical school deans and leadership, as well as respective class representatives, and by sending reminders for completion. The survey was not incentivized, and a higher response rate in the class of 2021 across all schools may indicate that students who felt their education was most affected were most likely to respond. We addressed this potential source of bias in the secondary analysis, which showed no differences between 2021 and 2022 respondents. Another limitation was the inherent issue with survey data collection of missing responses for some questions that occurred in a small number of surveys. This resulted in slight variability in the total responses received for certain questions, which were not statistically significant. To be transparent about this limitation, we presented our data by stating each total response and denominator in the Tables.

This initial study lays the groundwork for future investigations and next steps. With 72.1% of students agreeing that they were able to find meaningful learning in spite of the pandemic, future research should investigate novel learning modalities that were successful during this time. Educators should consider additional training on PPE use, given only moderate levels of student comfort in this area, which may be best received via video. It is also important to study the long-term effects of missing several months of essential clinical training and identifying competencies that may not have been achieved, since students perceived a significant disruption to their ability to prepare skills for residency. Next steps could be to study curriculum interventions, such as capstone boot camps and targeted didactic skills training, to help students feel more comfortable as they transition into residency. Educators must also acknowledge that some students may not feel comfortable returning to the clinical environment until a vaccine becomes available (5%) and ensure they are equally supported. Lastly, it is vital to further investigate the mental health effects of the pandemic on medical students, identifying subgroups with additional stressors, needs related to anxiety or possible PTSD, and ways to minimize these negative effects.

## Conclusions

In this cross-sectional survey, conducted during the initial peak phase of the COVID-19 pandemic, we capture a snapshot of the effects of the pandemic on US medical students and gain insight into their reactions to the unprecedented AAMC national recommendation for removal from clinical rotations. Student respondents from across the US similarly recognized a significant disruption to their medical education, shared a desire to continue with in-person rotations, and were willing to accept the risk of infection with COVID-19. Our novel results provide a solid foundation to help shape medical student roles in the clinical environment during this pandemic and future outbreaks.

## Supplementary Information

**Additional file 1: Table S1.** Survey Instrument

## Data Availability

The datasets used and/or analyzed during the current study are available from the corresponding author on reasonable request.

## References

[1] Association of American Medical Colleges. Interim Guidance on Medical Students’ Participation in Direct Patient Contact Activities: Principles and Guidelines. https://www.aamc.org/news-insights/press-releases/important-guidance-medical-students-clinical-rotations-during-coronavirus-covid-19-outbreak. Published March 17, 2020. Accessed April 1, 2020.

[2] Clark J (2003). Fear of SARS thwarts medical education in Toronto. BMJ..

[3] Loh LC, Ali AM, Ang TH, Chelliah A (2006). Impact of a spreading epidemic on medical students. Malays J Med Sci.

[4] Mortelmans LJ, Bouman SJ, Gaakeer MI, Dieltiens G, Anseeuw K, Sabbe MB (2015). Dutch senior medical students and disaster medicine: a national survey. Int J Emerg Med.

[5] Huapaya JA, Maquera-Afaray J, García PJ, Cárcamo C, Cieza JA (2015). Conocimientos, prácticas y actitudes hacia el voluntariado ante una influenza pandémica: estudio transversal con estudiantes de medicina en Perú [Knowledge, practices and attitudes toward volunteer work in an influenza pandemic: cross-sectional study with Peruvian medical students]. Medwave.

[6] Herman B, Rosychuk RJ, Bailey T, Lake R, Yonge O, Marrie TJ (2007). Medical students and pandemic influenza. Emerg Infect Dis.

[7] Kahn MJ, Markert RJ, Johnson JE, Owens D, Krane NK (2007). Psychiatric issues and answers following hurricane Katrina. Acad Psychiatry.

[8] Al-Rabiaah A, Temsah MH, Al-Eyadhy AA (2020). Middle East respiratory syndrome-Corona virus (MERS-CoV) associated stress among medical students at a university teaching hospital in Saudi Arabia. J Infect Public Health.

[9] Wong JG, Cheung EP, Cheung V (2004). Psychological responses to the SARS outbreak in healthcare students in Hong Kong. Med Teach.

[10] Stokes DC (2020). Senior medical students in the COVID-19 response: an opportunity to be proactive. Acad Emerg Med.

[11] Rodriguez RM, Medak AJ, Baumann BM (2020). Academic emergency medicine physicians’ anxiety levels, stressors, and potential mitigation measures during the acceleration phase of the COVID-19 pandemic. Acad Emerg Med.

[12] Sahu P (2020). Closure of universities due to coronavirus disease 2019 (COVID-19): impact on education and mental health of students and academic staff. Cureus.

[13] Reimers FM, Schleicher A. A framework to guide an education response to the COVID-19 pandemic of 2020: OECD. https://www.hm.ee/sites/default/files/framework_guide_v1_002_harward.pdf.

[14] Choi B, Jegatheeswaran L, Minocha A, Alhilani M, Nakhoul M, Mutengesa E (2020). The impact of the COVID-19 pandemic on final year medical students in the United Kingdom: a national survey. BMC Med Educ.

[15] Dyrbye LN, Thomas MR, Shanafelt TD (2006). Systematic review of depression, anxiety, and other indicators of psychological distress among U.S. and Canadian medical students. Acad Med.

