# Supplementary material for: Effects of the COVID-19 pandemic on medical students: a multicenter quantitative study
Source: BMC Med Educ. 2021 Jan 6;21:14. doi: 10.1186/s12909-020-02462-1 (PMC7786337; doi:10.1186/s12909-020-02462-1)
Supplement: Supplementary file 1 — Additional file 1: Table S1. Survey Instrument [file 12909_2020_2462_MOESM1_ESM.docx]

**Supplemental: Survey Instrument**

**Effects of the COVID-19 Pandemic on Medical Students: a Multicenter Quantitative Study**

*Thank you for agreeing to give your thoughts on the COVID-19 pandemic. In the first section, please consider the impact that the COVID-19 pandemic has had on you personally and professionally.*

*This survey is only for senior medical students who have started clinical rotations (class of 2020, 2021, or 2022).*

*There are 4 sections in this survey. It should take you 10-15 minutes to complete.*

1a. Have you had any direct patient care/contact in clinical settings since February 15? Y/N

1b. Are you currently on a clinical rotation with in-person patient care/contact? Y/N

1c. In which of these settings have you had in-person patient contact? (select all that apply)

- - Outpatient clinic
  - Emergency department
  - Inpatient hospital (non-ICU)
  - Intensive care unit
  - OR/Procedural suite
  - Other: ______________________

2a. Were you part of a clinical rotation that was cut short or cancelled due to the COVID-19 pandemic? Y/N

2b. If yes, what was your reaction to having the rotation cut short? (select all that apply)

- - - I felt relieved
    - I felt this was appropriate given the situation
    - I was disappointed and would have preferred to continue the rotation
    - I felt guilty for not being able to help patients and colleagues
    - I felt disrespected or devalued as part of the healthcare team
    - My reaction was neutral
    - Other: _____________________

2c. If you feel comfortable, please share some of the reasons for your reaction(s) above: _________________

3. Have you been part of a team taking care of patients suspected of having COVID-19? Y/N

4. How much has the COVID-19 pandemic affected your stress or anxiety levels?

| 1  Not at all | 2 | 3 | 4  Somewhat | 5 | 6 | 7  Extremely |
| --- | --- | --- | --- | --- | --- | --- |

5a. To what extent are you experiencing the following worries or concerns related to COVID-19?

| 1  Not at all | 2 | 3 | 4  Somewhat | 5 | 6 | 7  Extremely |
| --- | --- | --- | --- | --- | --- | --- |

5a_1. I worry that I may be or have been exposed in clinical or school-related activities

5a_2. I worry that as a healthcare worker, I may be secondarily exposing family members or others

5a_3. I worry that others at home or elsewhere are afraid to come in contact with me because I’m a healthcare provider

5a_4. I worry that I may have to self-quarantine at home

5a_5. I worry that I will not be able to get food and other necessities for me and my household

5a_6. I worry about my personal safety at home (domestic violence or abuse) during social distancing

5a_7. I worry about my own mental health at this time

5b. To what extent are you experiencing the following worries or concerns related to COVID-19?

| 1  Not at all | 2 | 3 | 4  Somewhat | 5 | 6 | 7  Extremely |
| --- | --- | --- | --- | --- | --- | --- |

5b_1. I worry that our hospital or clinic is not sufficiently prepared for the pandemic

5b_2. I worry that personal protective equipment (PPE) available in the hospital or clinic is inadequate

5b_3. I worry about the well-being of friends, family, and colleagues who have been diagnosed with COVID-19

5b_4. I worry that testing for COVID-19 has been inadequate

5b_5. I worry that undiagnosed, asymptomatic, or presymptomatic individuals are exposing others in the community

5b_6. I worry about the ethical decisions that may need to be made during the COVID-19 pandemic

5b_7. I worry about racial or other disparities in testing, treatment, and outcomes of COVID-19

6. To what extent were you experiencing severe, ongoing stress where you felt emotionally exhausted, burned out, cynical about medical school work and fatigued, even when you wake up...

| 1  Not at all | 2 | 3 | 4  Somewhat | 5 | 6 | 7  Extremely |
| --- | --- | --- | --- | --- | --- | --- |

6_a. … before the COVID-19 pandemic

6_b. … since the pandemic started

7.The following questions are to understand possible changes in your sleep related to the COVID-19 pandemic.

7a. How many hours of sleep have you gotten per night, on average, this week?

| 4  Or less | 5 | 6 | 7 | 8 | 9 | 10  Or more |
| --- | --- | --- | --- | --- | --- | --- |

7b. Is this more, the same, or less than you were sleeping on average before the start of the pandemic?

| More | The same | Less | Unsure |
| --- | --- | --- | --- |

7c. How much trouble have you had falling asleep or staying asleep over the past week?

| 1  No trouble at all | 2 | 3 | 4  Some trouble | 5 | 6 | 7  Extreme trouble |
| --- | --- | --- | --- | --- | --- | --- |

7d. How rested have you felt upon waking, on average, this week?

| 1  Not at all rested | 2 | 3 | 4  Somewhat rested | 5 | 6 | 7  Fully rested |
| --- | --- | --- | --- | --- | --- | --- |

8. Since the start of the COVID-19 pandemic, have you: (Y/N)

8_1. Had nightmares related to the pandemic or thought about the pandemic when you did not want to?

8_2. Tried hard not to think about the pandemic or gone out of your way to avoid situations that reminded you of it?

8_3. Been constantly on guard, watchful, or easily startled?

8_4. Felt numb or detached from people, activities or your surroundings?

8_5. Felt guilty or unable to stop blaming yourself or others for the effects of the pandemic or any problems the pandemic may have caused?

9. How often have you been bothered by the following over the past 2 weeks:

| Not at all | Several days | More than half the days | Nearly every day |
| --- | --- | --- | --- |

9_1. Feeling nervous anxious or on edge?

9_2. Not being able to stop or control worrying?

9_3. Worrying too much about different things?

9_4. Trouble relaxing?

9_5. Being so restless that it's hard to sit still?

9_6. Becoming easily annoyed or irritable?

9_7. Feeling afraid as if something awful might happen?

10. Where do you go to get most of your information on COVID-19? (select all that apply)

- - Social Media (Facebook, Twitter, Instagram, WhatsApp, etc.)
  - University communications or forums
  - Rotations (clinical or virtual)
  - Scientific publications (NEJM, JAMA, etc.)
  - Journalistic publications (NY times, Wall Street Journal, etc.)
  - Television or radio news
  - Other TV or radio (comedy shows, talk shows, etc.)
  - Friends and family
  - I try not to follow news about the pandemic
  - Other: _______________________________

*In this section, please consider what you know about the COVID-19 pandemic.*

11. How likely do you think it is that you may contract COVID-19?

| Extremely unlikely | Moderately unlikely | Slightly unlikely | Neither likely nor unlikely | Slightly likely | Moderately likely | Extremely likely |
| --- | --- | --- | --- | --- | --- | --- |

12. If you were to contract COVID-19, how likely do you believe each of these outcomes would be?

| Extremely unlikely | Moderately unlikely | Slightly unlikely | Neither likely nor unlikely | Slightly likely | Moderately likely | Extremely likely |
| --- | --- | --- | --- | --- | --- | --- |

12_1. Minimal impact, will not miss school or work

12_2. Will miss some school or work

12_3. Hospitalization but will recover

12_4. Death

13a. Have you changed your behavior (beyond what is recommended to the general public) with friends and family as a result of your possible exposure as a healthcare worker during the pandemic?

| Yes | No | Not applicable (no in-person patient contact |  |
| --- | --- | --- | --- |

13b. If yes, how have you changed your behavior? ____________________________________________

14a. How prepared do you feel to use personal protective equipment (PPE) to protect yourself from COVID-19 in the clinical setting?

| 1  Not at all | 2 | 3 | 4  Somewhat | 5 | 6 | 7  Extremely |
| --- | --- | --- | --- | --- | --- | --- |

14b. What gaps, if any, do you think exist in your knowledge and preparation to protect yourself from COVID-19 exposure in the clinical setting? (for example, when to wear masks, how to social distance in the clinical environment, etc.) _______________________________________________________

14c. What do you think would be the best method(s) to learn about PPE and other infection control measures during this pandemic? (select all that apply)

- - Online modules
  - In-person or Zoom conferences
  - Lab simulation
  - Video demonstration
  - PowerPoint
  - Newsletter or email
  - Journal article
  - Other: ________________________

15. How confident would you feel in identifying symptoms most concerning for COVID-19 in the clinical setting?

| 1  Not at all | 2 | 3 | 4  Somewhat | 5 | 6 | 7  Extremely |
| --- | --- | --- | --- | --- | --- | --- |

16. If there is not a COVID-19 vaccine until 2021, what would make you most comfortable returning to in-person patient care (on a clinical rotation or as an intern) in summer 2020? (select one)

- - Adequate testing for COVID-19 infection
  - Antibody testing for possible immunity to COVID-19
  - Adequate PPE for all healthcare workers and medical students
  - Data on how healthcare workers get infected at work
  - Nothing will make me feel comfortable until there is a vaccine
  - Other: ­­­­­­­­­­­­­­­­­­­­­­­­­­­­­­­­­­­­­­­­­­­­­­­­­­­________________________________

*In this section, please evaluate the impact that the COVID-19 pandemic has had on your medical education.*

17. To what extent do you agree or disagree with the following statements:

| Strongly disagree | Disagree | Somewhat disagree | Neither agree nor disagree | Somewhat agree | Agree | Strongly agree |
| --- | --- | --- | --- | --- | --- | --- |

17_1. To the extent possible, medical students should continue with normal clinical rotations during this pandemic.

17_2. Medical students should be allowed to volunteer in clinical settings during this pandemic, even if there is no healthcare worker shortage.

17_3. In the event of a healthcare worker shortage, medical students should be encouraged to volunteer clinically.

17_4. In the event of a healthcare worker shortage, medical students should be required to help clinically.

17_5. Medical students have a moral, ethical, or professional obligation to volunteer in the clinical setting during pandemics in general.

17_6. Medical students have a moral, ethical, or professional obligation to volunteer in the clinical setting during the COVID-19 pandemic.

17_7. The same obligation for medical students still applies during this pandemic, even if there is inadequate personal protective equipment.

17_8. Physicians (regardless of specialty) have a moral, ethical, or professional obligation to provide care in the clinical setting during pandemics in general.

17_9. Physicians (regardless of specialty) have a moral, ethical, or professional obligation to provide care in the clinical setting during the COVID-19 pandemic.

17_10. The same obligation for physicians applies during this pandemic, even if there is inadequate personal protective equipment.

18. If called upon to volunteer in a clinical environment at your current medical institution due to a healthcare worker shortage, which of these duties would you be willing to perform? (select all that apply)

- - Patient education
  - Telehealth
  - Screening visitors
  - Taking vital signs
  - Assisting with triage
  - Interviewing patients
  - Examining patients
  - COVID-19 testing
  - Low-risk procedures (IVs, suturing)
  - High-risk procedures (aerosolizing procedures like intubation)
  - Other: ___________________________

19. To what extent do you agree or disagree with the following statements:

| Strongly disagree | Disagree | Somewhat disagree | Neither agree nor disagree | Somewhat agree | Agree | Strongly agree |
| --- | --- | --- | --- | --- | --- | --- |

19_1. My medical education has been significantly disrupted by the pandemic.

19_2. I have been able to find meaningful learning opportunities in spite of the pandemic.

19_3. COVID-19 has shaped or influenced how I imagine spending my career.

19_4. I have felt connected with colleagues and classmates during the pandemic.

19_5. I feel supported by faculty, staff, and advisors in adjusting to changes brought on by the pandemic.

19_6. I believe that my medical school is doing everything they can to help students adjust.

19_7. The pandemic has interfered with my ability to apply to residency.

19_8. The pandemic has limited my ability to prepare competencies or skills I will need to start residency.

19_9. The pandemic has interfered with my intended graduation timeline.

19_10. I accept the risk that I may be infected with COVID-19 if I return to the clinical setting in 2020.

20. If you said above that you have been able to find meaningful learning experiences during the pandemic, what have been the most meaningful so far? _____________________________________________

21. What measures do you think would improve or have improved your educational experience during the pandemic? (select all that apply)

- - Lectures on COVID-19
  - Lectures on other clinical topics
  - Problem-based learning
  - Simulation learning
  - Structured distance learning (online courses)
  - Non-clinical skills practice (suturing, ultrasound, etc.)
  - Peer teaching
  - Research or scholarly work
  - Telehealth participation
  - Volunteering clinically
  - Volunteering non-clinically
  - Other: ________________________

*Finally, please supply some information about yourself.*

22. Age

| 20-24 | 25-29 | 30-34 | 35-39 | 40-44 | 45+ |  |
| --- | --- | --- | --- | --- | --- | --- |

23. Gender Identity (select all that apply)

- - Male
  - Female
  - Trans male
  - Trans female
  - Genderqueer/Non-binary
  - Other: ____________________________

24. Race/Ethnicity (select all that apply)

- - African-American
  - Asian
  - Hispanic/Latinx
  - Native American/American Indian
  - Native Hawaiian or Pacific Islander
  - White (non-Latinx)
  - Other: ­­­­­­­­­­­­­­­­­­­­­­­­­­____________________________

25. What is your expected graduation year excluding any planned time off? (This study is intended for senior medical students in their clinical rotation years.)

| Class of 2020 | Class of 2021 | Class of 2022 |  |
| --- | --- | --- | --- |

26. Do you have anyone living with you in your home? (select all that apply)

- - Live alone
  - Roommate(s)
  - My partner(s)
  - Children <18 years old
  - My parent(s)
  - Friends
  - Someone > 70 years old
  - Someone else at higher risk for COVID-19 complications (heart or lung disease, diabetes, immunocompromised)
  - Other: ______________________

27a. Planned Specialty:

- - (choose one)
  - Anesthesiology
  - Dermatology
  - Emergency Medicine
  - Family Practice
  - General Surgery
  - Internal Medicine
  - Internal Medicine-Pediatrics
  - Neurology
  - Neurosurgery
  - Obstetrics/Gynecology
  - Ophthalmology
  - Orthopedic Surgery
  - Otolaryngology
  - Pathology
  - Pediatrics
  - Physical Medicine
  - Plastic Surgery
  - Psychiatry
  - Radiation Oncology
  - Radiology
  - Urology
  - Undecided

27b. Did you match into this specialty in March 2020?

- - Yes, I did match in this specialty
  - No, I did not match in this specialty
  - I did not enter 2020 Match

28. In which state do you attend medical school? _________________________

29. Which medical school do you attend?

- - UC San Francisco
  - UC Irvine
  - University of Illinois
  - Tulane
  - Hofstra
  - Ohio State University
  - Other: ___________________________________
